# Reciprocal modulation of responses to nitrate starvation and hypoxia in roots and leaves of *Arabidopsis thaliana*

**DOI:** 10.1080/15592324.2023.2300228

**Published:** 2024-01-02

**Authors:** Vajiheh Safavi-Rizi, Tina Uhlig, Felix Lutter, Hamid Safavi-Rizi, Franziska Krajinski-Barth, Severin Sasso

**Affiliations:** a Institute of Biology, Department of Plant Physiology, Leipzig University, Leipzig, Germany; b Institute of Biology, Department of General and Applied Botany, Leipzig University, Leipzig, Germany; c Department of Information Technology Engineering, Institute of Information Technology and Computer Engineering, University of Payame Noor, Isfahan, Iran

**Keywords:** Nitrogen deficiency, hypoxia, climate change, combined stress, *Arabidopsis thaliana*

## Abstract

The flooding of agricultural land leads to hypoxia and nitrate leaching. While understanding the plant’s response to these conditions is essential for crop improvement, the effect of extended nitrate limitation on subsequent hypoxia has not been studied in an organ-specific manner. We cultivated *Arabidopsis thaliana* without nitrate for 1 week before inducing hypoxia by bubbling the hydroponic solution with nitrogen gas for 16 h. In the roots, the transcripts of two transcription factor genes (*HRA1*, *HRE2*) and three genes involved in fermentation (*SUS4*, *PDC1*, *ADH1*) were ~10- to 100-fold upregulated by simultaneous hypoxia and nitrate starvation compared to the control condition (replete nitrate and oxygen). In contrast, this hypoxic upregulation was ~5 to 10 times stronger when nitrate was available. The phytoglobin genes *PGB1* and *PGB2*, involved in nitric oxide (NO) scavenging, were massively downregulated by nitrate starvation (~1000-fold and 10^5^-fold, respectively), but only under ambient oxygen levels; this was reflected in a 2.5-fold increase in NO concentration. In the leaves, *HRA1*, *SUS4*, and *RAP2.3* were upregulated ~20-fold by hypoxia under nitrate starvation, whereas this upregulation was virtually absent in the presence of nitrate. Our results highlight that the plant’s responses to nitrate starvation and hypoxia can influence each other.

## Introduction

The world population is predicted to exceed 9 billion by the 2050s, and crop production may need to be increased by more than 85% compared to the levels of 2013.^1^ In agriculture, nitrogen is a limiting factor for plant growth and is essential for the biosynthesis of nucleic acids, amino acids, chlorophyll, many vitamins and secondary metabolites, and it contributes to the response to various biotic and abiotic stresses.^2^ Nitrogen limitation in crops is compensated by fertilization. However, the application of excess fertilizer has drastic ecological consequences such as eutrophication and unnecessary emission of greenhouse gases into the atmosphere, intensifying global warming.^3^

Global warming leads to frequent flooding periods that compromise crop yields.^4^ Floodings are estimated to have resulted in crop losses of 5.5 billion United States dollars from 1982 to 2016.^5^ Under flooding, the slower diffusion of oxygen in water compared to air leads to low environmental oxygen concentrations (hypoxia).^6^ Decreasing energy supply due to limiting mitochondrial respiration is one of the major events during hypoxia which induces alternative metabolic activities such as glycolysis and fermentation for energy production.^7–9^ Mutations in genes for key enzymes involved in ethanolic fermentation, such as pyruvate decarboxylase (PDC) or alcohol dehydrogenase (ADH), decrease plant tolerance for submergence and hypoxia.^10,11^ In flooded plants, ethylene entrapment is essential for the acclimation to hypoxia, and ethylene-mediated homeostasis of nitric oxide (NO) and reactive oxygen species (ROS) is indispensable for hypoxia tolerance.^12–14^ The prerequisite of a proper hypoxia response is an efficient mechanism of oxygen sensing and signaling. In *Arabidopsis thaliana*, oxygen sensing and signaling is conducted via the oxygen- and NO-dependent N-degron pathway that leads to the proteasomal degradation of subgroup VII ethylene response factors (ERFVII) under aerobic conditions. When stabilized under hypoxic conditions, the ERFVII transcription factors translocate from the cytosol to the nucleus and induce the expression of hypoxia response genes.^15,16^

In the field, plants are often exposed to multiple types of stress and have developed various physiological and molecular mechanisms to cope with such situations.^17^ Several lines of evidence suggest that nitrogen metabolism may be involved in the cellular response to hypoxia.^7–21^ NO represents a plausible link between hypoxia and nitrate levels. NO can be formed by nitrate reductase and appears to participate in the response of maize roots to nitrate, particularly in the root transition zone, a highly nitrate-responsive region.^22,23^ In poplar roots, hypoxia induces the formation of NO in a nitrate-dependent manner.^24^ However, our knowledge of the effects of extended nitrogen deficiency on the hypoxia tolerance in different organs is still scarce. This study examines how nitrate starvation affects the response of leaves and roots to hypoxia in *A. thaliana*. We assessed several physiological parameters and quantified the transcript levels of marker genes involved in nitrogen deficiency and hypoxia. The results illuminate a dynamic interplay between nitrogen levels and hypoxia in an organ-specific manner.

## Materials and methods

### Plant material and growth conditions

To sterilize the surface of *A. thaliana* (Col-0) seeds, approximately 20 mg of seeds were incubated in a solution of 7 g of Ca(ClO)₂ dissolved in 10 ml of 37% hydrochloric acid in a closed desiccator under a fume hood for 7 h. Seeds were germinated on sterile 0.7% agar in the growth room with 150 μmol photons m^−2^ s^−1^ and 25°C under a 16/8-h light/dark regime. After 1 week, several seedlings were transferred to the hydroponic boxes containing 2.3 L of 80% nutrient solution. The nutrient solution (100%) contained 5 mM KNO_3_, 1.5 mM Ca(NO_3_)_2_, 1 mM NH_4_Cl, 1 mM MgSO_4_, 1 mM KH_2_PO_4_, 79.5 μM Fe(III)EDTA, 46 μM H_3_BO_3_, 0.8 μM ZnSO_4_, 0.32 μM CuSO_4_, 0.01 μM CoCl_2_, 0.6 μM Na_2_MoO_4_, 10 μM MnSO_4_, 0.25 μM NH_4_VO_3_) bubbled with air, according to.^25^ In addition, 2 mM MES buffer, pH 5.7, 0.1 mM K_2_SiO_3_^26^ and 0.01 mM CoCl_2_^27^ was used in the 100% nutrient solution. The roots were in contact with the medium. The medium was renewed every other day for another 2 weeks before the stress treatment.

### Induction of nitrate starvation and hypoxia

Three-week old plants were divided into two groups: the control (+NO_3_^−^: nutrient solution with 6.4 mM nitrate and 1 mM ammonium) and the nitrate starvation group (-NO_3_^−^: only 1 mM ammonium). For nitrate starvation, all nitrate salts were substituted by the corresponding chloride salts. Plants were grown for another week under these conditions, and the medium was renewed every other day. For the hypoxia treatment, 4-week-old plants from the +NO_3_^−^ and -NO_3_^−^ treatments were further divided into two groups each: oxygen-replete conditions (+NO_3_^−^/+O_2_, -NO_3_^−^/+O_2_) and hypoxia (+NO_3_^−^/-O_2_, -NO_3_^−^/-O_2_). The nitrate starvation treatment was continued for -NO_3_^−^ plants during the hypoxia period. Hypoxia was induced by bubbling the nutrient solution with N_2_ gas (≥99.99 vol. %) (Air Liquide, Germany) with an approximate flow rate of 14 L h^−1^, starting at 4.00 p.m. Hypoxia was induced in the dark to avoid the photosynthetic generation of oxygen. To prevent differences in the light conditions between different treatments all of the plants were kept in the dark. The oxygen concentration of the nutrient solution was measured after 16 h of hypoxia (at 8.00 a.m. on the next day) using a Multimeter Multi 340i oxygen electrode (WTW GmbH, Germany) following the manufacturer’s instructions before the rosette leaves and roots were harvested.

### Measurements of dry weight and chlorophyll content

Leaves and roots were put in coffee filter bags and dried in a drying cabinet (Heraeus, Heraeus Function Line) at 78°C for 48 h. The dry weight was measured using an analytical balance (BP 210 D, Sartorius).

The relative chlorophyll levels of fully developed leaves were determined using a SPAD-502 chlorophyll meter (Konica Minolta, Japan). For each biological replicate, SPAD values from three similar positions on a leaf were recorded, and their average was calculated.

### RNA isolation, cDNA synthesis, and real-time PCR

For reverse transcription-quantitative real-time PCR (RT-qPCR) analysis, total RNA was extracted from frozen leaves and roots using the NucleoSpin RNA plant and Fungi kit (Macherey-Nagel, Germany) according to the manufacturer’s instructions. RNA concentration and purity were quantified using a NanoDrop ND-1000 photospectrometer (Thermo Scientific, Germany). RNA integrity was confirmed on a 1.2% agarose gel. DNase I treatment was conducted on 2 μg RNA. Complementary DNA (cDNA) was synthesized using the RevertAid H Minus First Strand kit (Thermo Scientific) with oligo-(dT)_18_ primers. After elution in 20 μl RNase free water samples were subsequently diluted by adding 20 μl deionized water to the final volume of 40 μl. RT-qPCR reactions were performed in a total reaction volume of 5 μl containing 2.5 μl Power SYBR Green Master Mix (Thermo Scientific), 0.5 μM forward primer, 0.5 μM reverse primer, and 0.5 μl of cDNA. *ACTIN2* was used as a reference gene. The thermal profile used for all RT-qPCRs was: 2 min 95°C; 40 cycles of (3 s 95°C; 30 s 60°C); the data were analyzed by the 2^−ΔΔCt^ method.^28^ Specific Primers for the genes involved in nitrogen assimilation and NO scavenging were designed using Quantprime software.^29^ All sequences of gene-specific primers are provided in Supplementary Table S1.

### NO quantification

For the quantification of intracellular NO, the fluorescence dye 4-amino-5-methylamino-2‘,7’-difluorofluoresceine diacetate (DAF-FM diacetate, Sigma-Aldrich), was used.^30^ For each treatment, the roots were separated from the leaves, and 1 cm of the root tip, including lateral roots, was cut and incubated in 10 mM MES-Tris buffer, pH 7.0, containing 10 µM DAF-FM diacetate under agitation (350 rpm) at 23°C for 30 min. To protect the hypoxia-treated plants from oxygen exposure, the nutrient solution was bubbled with N_2_ gas during the harvesting process (approximately 5 min). Moreover, the buffer-DAF-FM diacetate solution was bubbled with N_2_ gas for approximately 30 s before the roots were added, and the bubbling was continued during incubation. The roots were washed twice with the MES-Tris buffer for 30 s before being mounted on a microscope slide. The same buffer solution was used as the mounting solution. To visualize the fluorescence signal, a Zeiss LSM700 Axio Observer with excitation at 488 nm and emission between 515 and 580 nm, and a Plan-Apochromat 20×/0.8 M27 objective was used. To determine the background signal, the same incubation was performed without DAF-FM diacetate, and this signal was subtracted from the signal with DAF-FM diacetate. The settings of the digital gain, pinhole, laser power and detector offset were kept constant for all measurements. Using the ZEN (blue edition) software (Carl Zeiss Microscopy GmbH), the mean fluorescence pixel intensity was quantified in similar areas of approximately 17,000 µm^2^ of the root tip.^14^

### Statistical analysis

Significant differences were calculated by using two-way ANOVA using GraphPad Prism software (v. 9.4.1) to examine the effects of nitrate (NO_3_^−^) and oxygen (O_2_). A Tukey’s multiple-comparison test was used to compare all growth conditions.

## Results and discussion

### Effects of nitrate starvation and hypoxia on growth and physiological activity

**Figure 1.**
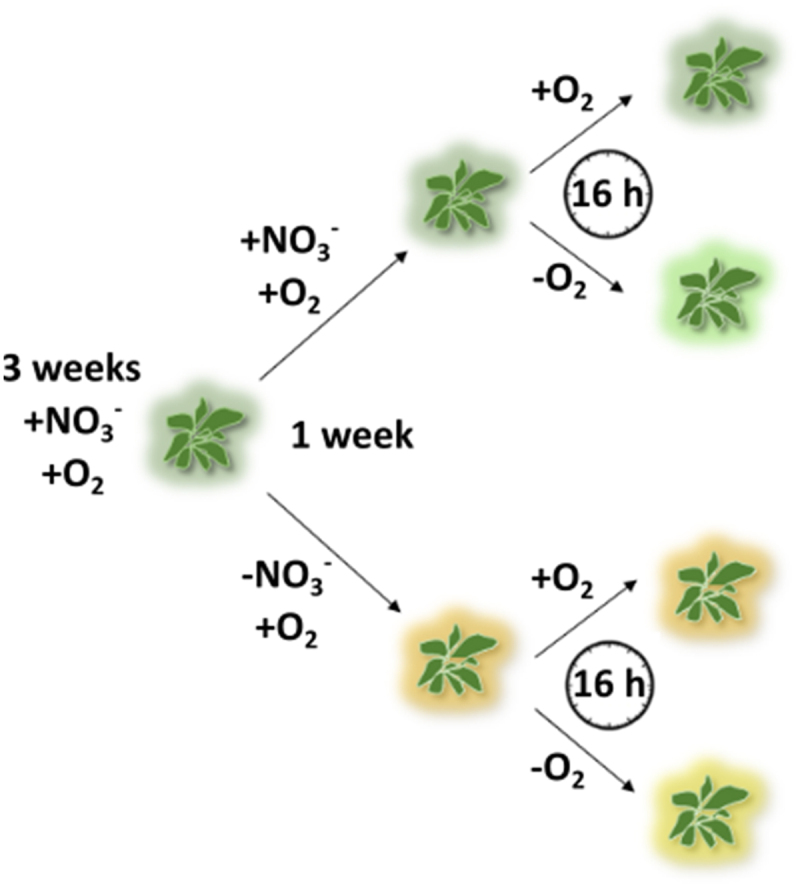
Overview of the experimental set-up. *A. thaliana* plants were grown hydroponically for 3 weeks under nitrate- and oxygen-replete conditions (+NO_3_^−^/+O_2_). Afterwards, the plants were divided into two groups receiving either adequate nitrate (+NO_3_^−^) or nitrate starvation (-NO_3_^−^). After one week, each group was further divided in two subgroups, oxygen-replete (+NO_3_^−^/+O_2_ and -NO_3_^−^/+O_2_) or hypoxia (+NO_3_^−^/-O_2_ and -NO_3_^−^/-O_2_), and incubated for another 16 h.

To investigate the effect of nitrate starvation on the hypoxia response in the leaves and roots of *A. thaliana*, 3-week old hydroponically grown plants were provided with either 6.4 mM (+NO_3_^−^) or no nitrate (-NO_3_^−^) (Figure 1). In both cases, the nutrient solution was bubbled with air and contained 1 mM ammonium. After 1 week, half of the plants were subjected to 16 h of hypoxia (-O_2_) by changing the bubbling to N_2_, while the bubbling with air (+O_2_) was continued for the other half of the plants (Figure 1). At the end of this treatment, the concentration of dissolved oxygen around the roots was (1.0 ± 0.2) mg L^−1^ for the samples bubbled with N_2_ and (4.4 ± 0.1) mg L^−1^ for the samples bubbled with air.

**Figure 2.**
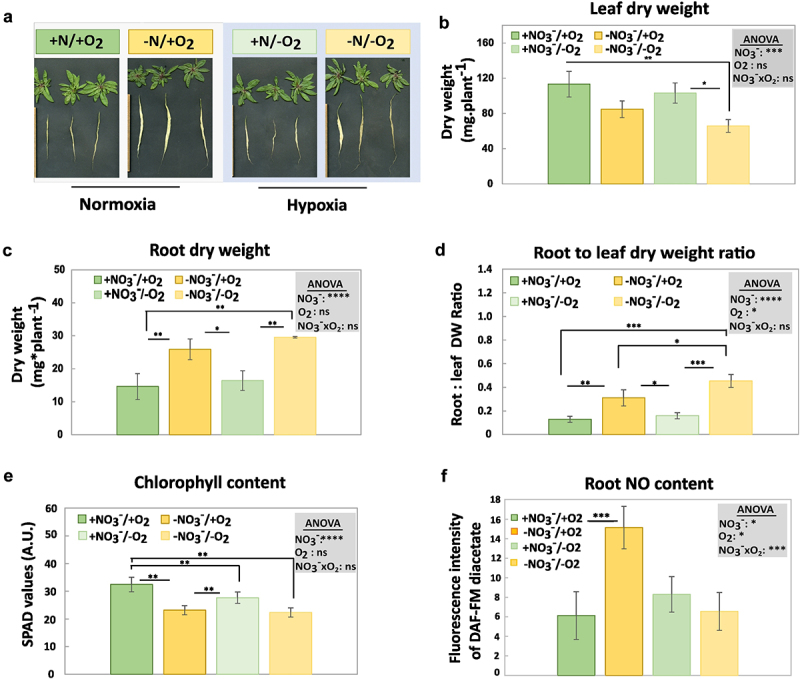
Effects of nitrate starvation and hypoxia on the phenotype and physiological parameters of *A. thaliana*. A) phenotype of plants with dissected leaves and roots, B) leaf dry weight, C) root dry weight, D) root to leaf dry weight ratio, E) leaf chlorophyll content, F) NO content of the root tip and lateral root (1 cm). NO_3_^−^/O_2_ treatments are described in Figure 1. Error bars represent standard deviations calculated from biological replicates (*n* = 3 in B-E, *n* = 4 in F). Grey boxes represent statistical analysis by 2-way ANOVA: +NO_3_^−^, nitrate; O_2_, oxygen; NO_3_^−^xO_2_, interactions between nitrate and oxygen. Significance levels are indicated as ns *p* > 0.05, * *p* < 0.05, ** *p* < 0.01, *** *p* < 0.001, and **** *p* < 0.0001. Subsequently, a Tukey’s HSD post hoc test was performed to identify significant differences between all different treatments (multiple comparison), represented as stars on the bars.

To assess the effect of nitrate starvation and hypoxia on *A. thaliana*, we examined several phenotypic and physiological traits (Figure 2). Compared to plants grown under replete nitrate and oxygen (defined as the control condition), nitrate starvation decreased the dry weight of leaves under hypoxia and increased the length and dry weight of roots (Figures 2a–c). While no significant effect of hypoxia on these traits was detected compared to the control, hypoxia further amplified the increase in the root-to-shoot ratio caused by nitrate deficiency by 1.7-fold (Figure 2d). Chlorophyll content decreased in the leaves of nitrate-limited plants compared to the control, whereas hypoxia only provoked a small decrease in chlorophyll content under replete nitrate (Figure 2e). NO content was 2.5 times higher under nitrate starvation compared to the control (Figure 2f). In contrast, the NO levels were unaffected by hypoxia or combined hypoxia and nitrate starvation (Figure 2f), suggesting that hypoxia suppresses the increase in NO levels induced by limiting nitrate.

Taken together, the phenotypic and physiological traits were mainly influenced by nitrate availability, and the duration of hypoxia was probably too short to cause major phenotypic or physiological changes. Nevertheless, our results on root-to-shoot ratios and NO content demonstrate that the response to nitrate starvation and the response to hypoxia can mutually influence each other.

### Regulation of glycolysis and fermentation genes by nitrate starvation and hypoxia

Mitochondrial ATP production is impaired under hypoxia due to oxygen limitation leading to energy crisis for the cell.^6^ Therefore, under oxygen limitation, plants use glycolysis and fermentation to produce ATP and regenerate NAD^+.8^ In this study, the transcript levels of genes for sucrose synthase (*SUS4*), pyruvate decarboxylase (*PDC1*), and alcohol dehydrogenase (*ADH1*)^16^ were quantified by reverse transcription-quantitative real-time PCR (RT-qPCR) to explore how nitrate starvation and hypoxia affect fermentation.

**Figure 3.**
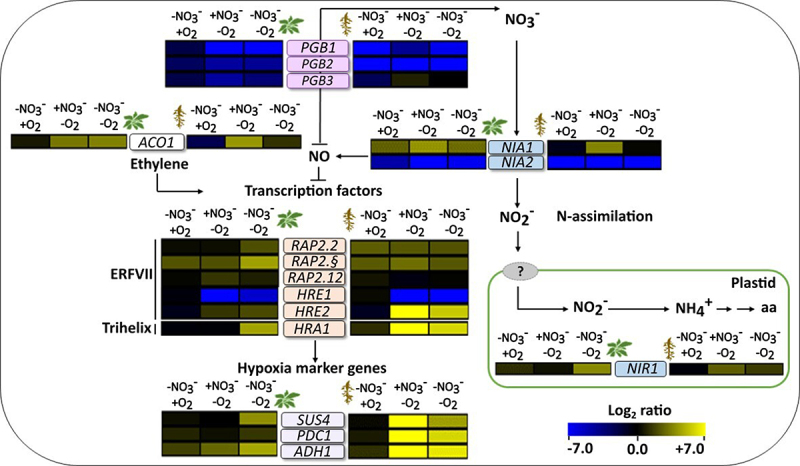
Effects of nitrate starvation and hypoxia on the transcript levels of marker genes. The color scale represents the log_2_ fold changes in transcript levels relative to the control condition (+NO_3_^−^/+O_2_), with the changes in leaves depicted on the left and the changes in roots depicted on the right of the gene names (blue, downregulated; yellow, upregulated). NO_3_^−^/O_2_, treatments are described in Figure 1. The data represent the means of three biological replicates. The heatmap was generated using the multiple experiment viewer (MeV) software (https://webmev.tm4.org). Please note that the arrows may not fully explain the observed expression patterns but rather illustrate the known links between the different genetic and metabolic components. Supplementary figure S1 shows the same data with additional statistical details. *SUS4, SUCROSE SYNTHASE 4; PDC1, PYRUVATE DECARBOXYLASE 1; ADH1, ALCOHOL DEHYDROGENASE 1; ACO1*, *1-AMINOCYCLOPROPANE-1-CARBOXYLATE OXIDASE*; *RAP2.2, RELATED to AP2 2; RAP2.3*, *RELATED to AP2 3; RAP2.12*, *RELATED to AP2 12; HRA1*, *HYPOXIA RESPONSE ATTENUATER 1; HRE1*, *HYPOXIA RESPONSIVE ERF (ETHYLENE RESPONSE FACTOR) 1*; *HRE2, HYPOXIA RESPONSIVE ERF (ETHYLENE RESPONSE FACTOR) 2*; *NIA1, NITRATE REDUCTASE 1; NIA2, NITRATE REDUCTASE 2; NIR1*, *NITRITE REDUCTASE 1*.

In the roots, all three genes were approximately 100- to 1000-fold upregulated by hypoxia under nitrate-replete conditions; this strong upregulation by hypoxia was attenuated when nitrate was lacking (Figure 3 and Supplementary Figure S1). In contrast, the three marker genes were only moderately (~10- to 20-fold) upregulated in the leaves, and only under combined hypoxia and nitrate starvation (Figure 3). These results clearly demonstrate that the response of *A. thaliana* to hypoxia is modulated by nitrogen availability. Moreover, pronounced differences were observed between leaves and roots.

### Regulation of ethylene biosynthesis and response genes by nitrate starvation and hypoxia

Nitrate starvation can transiently increase ethylene biosynthesis and signaling.^31^ We investigated how nitrate starvation affects the expression of the ethylene biosynthesis gene *ACO1*, encoding 1-aminocyclopropane-1-carboxylate oxidase, under hypoxia. *ACO1* exhibited upregulation under hypoxia and simultaneous hypoxia and nitrate starvation in the leaves, whereas in the roots, *ACO1* was upregulated only under hypoxia and replete nitrate (Figure 3).

ERFVII transcription factors are essential for the hypoxia response.^16^ For example, the hypoxia-induced ERFVII gene *RAP2.2* is important for hypoxia tolerance.^32,33^ Another ERFVII gene, *RAP2.12*, is modulated by the trihelix transcription factor gene *HRA1*, which is also induced by hypoxia.^10,34^ In our experiment, combined nitrate starvation and hypoxia led to a moderate upregulation (~5–20-fold) of *RAP2.2* and *RAP2.3* in the leaves and roots compared to the control condition (Figure 3). In contrast, the tested conditions did not result in any major changes in the transcript levels of *RAP2.12* (Figure 3). The *HRA1* transcript was moderately (~20-fold) upregulated under combined hypoxia and nitrogen starvation in leaves (Figure 3). In the roots, the hypoxia-induced upregulation of *HRA1* was strong under replete nitrogen (more than 500-fold), but less pronounced (~60-fold) in the absence of nitrate (Figure 3).

We finally tested two additional ERFVII transcription factor genes, *HRE1* and *HRE2*, which are known to be hypoxia-inducible.^35^ The transcript analysis revealed significant downregulation of the *HRE1* in the leaves, with a 500-fold decrease under hypoxia and a 50-fold decrease under combined nitrate starvation and hypoxia (Figure 3). Notably, the roots exhibited the most substantial downregulation, with a 1000-fold decrease in transcript levels both under hypoxia and combined stress. For *HRE2*, the regulation in the roots was remarkably similar to the patterns previously observed for *SUS4*, *PDC1*, *ADH1*, and *HRA1*: The *HRE2* transcript levels were strongly (~300-fold) increased under hypoxia when nitrate was available, whereas this upregulation was attenuated by nitrate starvation (Figure 3). Collectively, our data indicate that nitrate availability can strongly affect the regulation of the ethylene response genes under hypoxia.

### Regulation of nitrogen and NO metabolism genes by nitrate starvation and hypoxia

Nitrate reduction, the rate-limiting step in nitrogen assimilation, is catalyzed by nitrate reductase. This reaction is NADH-dependent and therefore associated with hypoxia acclimation through the regeneration of NAD^+^ from NADH.^18^ In our experiment, the nitrate reductase-encoding genes, *NIA1* and *NIA2*, were differentially modulated in response to nitrate starvation and hypoxia. In leaves, *NIA1* was upregulated under nitrate starvation, hypoxia and combined stress, while no significant changes in *NIA1* transcripts were observed in the roots (Figure 3). In contrast, *NIA2* was downregulated in response to nitrate starvation, hypoxia and a combination of both, with the strongest downregulation (~1000 fold) observed in the roots under combined nitrate starvation and hypoxia (Figure 3 and Supplementary Figure S1).

Nitrite reductase catalyzes the second step of nitrogen assimilation, the conversion of nitrite to ammonium.^36^ In the current study, the *NIR1* transcript was upregulated only under simultaneous nitrate starvation and hypoxia (Figure 3), indicating that the combination of these factors leads to differential modulation of *NIR1* transcription. These data further emphasize the importance of the nitrate status for the response to hypoxia.

NO metabolism is suggested to be one of the main functions of phytoglobins in plants.^37^ Under very low atmospheric oxygen concentrations (below 1%), the phytoglobin/NO cycle converts NO to nitrate.^38^ This cycle consumes NADH and NADPH providing NAD^+^ and NADP^+^ which is essential for ATP production via the glycolytic pathway.^39^

To explore how nitrate starvation and hypoxia affect the regulation of genes involved in NO scavenging, we measured the expression of the phytoglobin genes *PGB1*, *PGB2* and *PGB3*. Phytoglobin genes are induced under hypoxia, and an increased transcript level of *PGB1* improves hypoxia tolerance in *A. thaliana*.^40,41^ In the current study, all three genes were downregulated in leaves under hypoxia and combined nitrate starvation and hypoxia compared to the control condition (Figure 3). In the roots, the transcript levels of *PGB1* and *PGB2* were decreased under all treatments compared to the control. *PGB2* exhibited the strongest downregulation under nitrate starvation (more than 10^5^-fold), followed by hypoxia and combined nitrate starvation and hypoxia (~10^4^-fold each) (Figure 3). The nitrate starvation-induced downregulation of *PGB2* in the roots was in accordance with higher NO levels compared to nitrate-replete conditions (Figure 2f).

## Conclusion

This study reveals a critical relationship between nitrate availability and the hypoxia response in an organ-specific manner in *A. thaliana*. In our experiment, nitrate availability modulated the hypoxia-induced changes in transcript levels in several genes in the roots (e.g. *SUS4*, *PDC1*, *ADH1*) and in the leaves (e.g. *HRA1*) (Figure 3). On the other hand, oxygen availability affected the changes in *PGB1* and *PGB2* expression triggered by the lack of nitrate, similar to its influence on NO levels in the roots (Figure 3). The distinct responses of roots and leaves to different combinations of nitrate and oxygen availability emphasize the organ-specific nature of the plant’s response to multiple forms of stress. These findings contribute to a better understanding of how plants cope with nitrogen scarcity and hypoxia, which can be provoked by flooding due to global warming. A further exploration of the response to simultaneous types of stress in different plant species, including a detailed characterization of ethylene and NO signaling using appropriate mutants, will be important for enhancing stress resilience in crops.

## Supplementary Material

Supplementary Table S1.docx

Supplementary Figure S1.docx
