# Supplementary material for: Reciprocal modulation of responses to nitrate starvation and hypoxia in roots and leaves of Arabidopsis thaliana
Source: Plant Signal Behav. 2024 Jan 2;19(1):2300228. doi: 10.1080/15592324.2023.2300228 (PMC10763642; doi:10.1080/15592324.2023.2300228)
Supplement: Supplementary Table S1.docx [file KPSB_A_2300228_SM1262.docx]

**Supplementary Table S1. List and sequences of primers used in this study.**

| **Target gene** | **Gene ID** | **Primer name** | **Oligo sequence 5' → 3'** | **Reference** |
| --- | --- | --- | --- | --- |
| *ACO1* | AT2G19590 | ACO1_F | TGGAGATGCGTTATTGTGA | Hartman et al., 2019 |
|  |  | ACO1_R | GCGAGAAGGTAAGCGAAG |  |
| *HRA* | AT3G10040 | HRA_F | CATGACCAACAACCACCGCAAC |  |
|  |  | HRA_R | TTCTGCTGCTGACTCGGAATCG |  |
| *HRE1* | AT1G7236 | HRE1_F | TCCGATGAGCCATTTGTCTTCTCC |  |
|  |  | HRE1_R | CCATCTTCCCCAAGGCCTTC |  |
| *HRE2* | AT2G47520 | HRE2_F | TTGCTGCCATCAAAATCCGT |  |
|  |  | HRE2_R | CCCCTGGTTTAGTATCGGCT |  |
| *PDC1* | AT4G33070 | PDC1_F | TCGATTGGGTGGTCTGTTGG |  |
|  |  | PDC1_R | TGTCCTGAACCGTGACTTGG |  |
| *PDC2* | AT5G54960 | PDC2_F | TGAAAGCAATCAACACGGCA |  |
|  |  | PDC2_R | CAGCAGAGACTCTAGAGCCC |  |
| *SUS4* | AT3G43190 | SUS4_F | TTCACCATGGCTAGGCTTGA |  |
|  |  | SUS4_R | CCACCAAGTTCACCAGTTCG |  |
| *RAP2.2* | AT3G14230 | RAP2.2_F | CCTAGCGTCGTATCCCAGAA |  |
|  |  | RAP2.2_R | CTCAGATGTGTTGGCTGCTG |  |
| *RAP2.3* | AT3G16770 | RAP2.3_F | AACTCACGGCTGAGGAACTCTG |  |
|  |  | RAP2.3_R | ACGTTAACTTGGTTGGTGGGATGG |  |
| *RAP2.12* | AT1G53910 | RAP2.12_F | ACTGAATGGGACGCTTCACTGG |  |
|  |  | RAP2.12_R | AGGGTTTGCACCATTGTCCTGAG |  |
| *ADH1* | AT1G77120 | ADH1_F | GGTCTTGGTGCTGTTGGTTT |  |
|  |  | ADH1_R | CTCAGCGATCACCTGTTGAA |  |
| *PDC1* | AT4G33070 | PDC1_F | TCGATTGGGTGGTCTGTTGG |  |
|  |  | PDC1_R | TGTCCTGAACCGTGACTTGG |  |
| *PDC2* | AT5G54960 | PDC2_F | TGAAAGCAATCAACACGGCA |  |
|  |  | PDC2_R | CAGCAGAGACTCTAGAGCCC |  |
| *NIA1* | AT1G77760 | NIA1_F | CTGAGCTGGCAAATTCCGAAGC | This study |
|  |  | NIA1_R | TGCGTGACCAGGTGTTGTAATC |  |
| *NIA2* | AT1G37130 | NIA2_F | AACTCGCCGACGAAGAAGGTTG |  |
|  |  | NIA2_R | GGGTTGTGAAAGCGTTGATGGG |  |
| *NIR1* | AT2G15620 | NIR1_F | TGTGGCTTATCGACGAACTTGGTG |  |
|  |  | NIR1_R | CCTCTCTCGAGTTTCCCATTTGGC |  |
| *PGB1* | AT2G16060 | PGB1_F | AGAGACTTGGAGCCAGCCATTC |  |
|  |  | PGB1_R | ACAATGCATACTTGGCCACCTC |  |
| *PGB2* | AT3G10520 | PGB2_F | AGGAAAGGTGGTAGTGGCTGAC |  |
|  |  | PGB2_R | TAGCAAAGCTTCTTTCACCACCTC |  |
| *PGB3* | AT4G32690 | PGB3_F | CCGCATCTAGATCGCTACCAAACC |  |
|  |  | PGB3_R | CGGCGTGAACTTTGTCCTGAAC |  |
| *ACTIN 2* | AT2G37620 | ACTIN 2_F | TCCCTCAGCACATTCCAGCAGAT | González et al., 2007 |
|  |  | ACTIN 2_R | AACGATTCCTGGACCTGCCTCATC |  |
