# Supplementary material for: Reciprocal modulation of responses to nitrate starvation and hypoxia in roots and leaves of Arabidopsis thaliana
Source: Plant Signal Behav. 2024 Jan 2;19(1):2300228. doi: 10.1080/15592324.2023.2300228 (PMC10763642; doi:10.1080/15592324.2023.2300228)
Supplement: Supplementary Figure S1.docx [file KPSB_A_2300228_SM1261.docx]

**Supplementary Figure S1. Effect of nitrogen deficiency and/or hypoxia on the transcript levels of the investigated genes in this study, in the roots and leaves.** All expression fold changes are calculated relative to control (+N/+O_2_)**.** Gene expression was measured using real-time PCR. The control expression level is set to 1 and the expression fold changes are shown relative to control. Growth conditions are shown in the legend: +N/+O_2_, control; -N/+O_2_, one week without nitrate/normoxia; +N/-O_2_, normal nitrate supply/16 h hypoxia; -N/-O_2_, one week without nitrate/16 h hypoxia(double stress). Statistical analysis was performed by 2-way ANOVA: N, nitrate; O_2_, oxygen; NxO_2_, interactions between nitrate and oxygen. Significance levels are indicated as ns p>0.05, * p<0.05, ** p<0.01, *** p<0.001, and **** p<0.0001. 3 biological replicates were used. Error bars represent standard deviation.

**
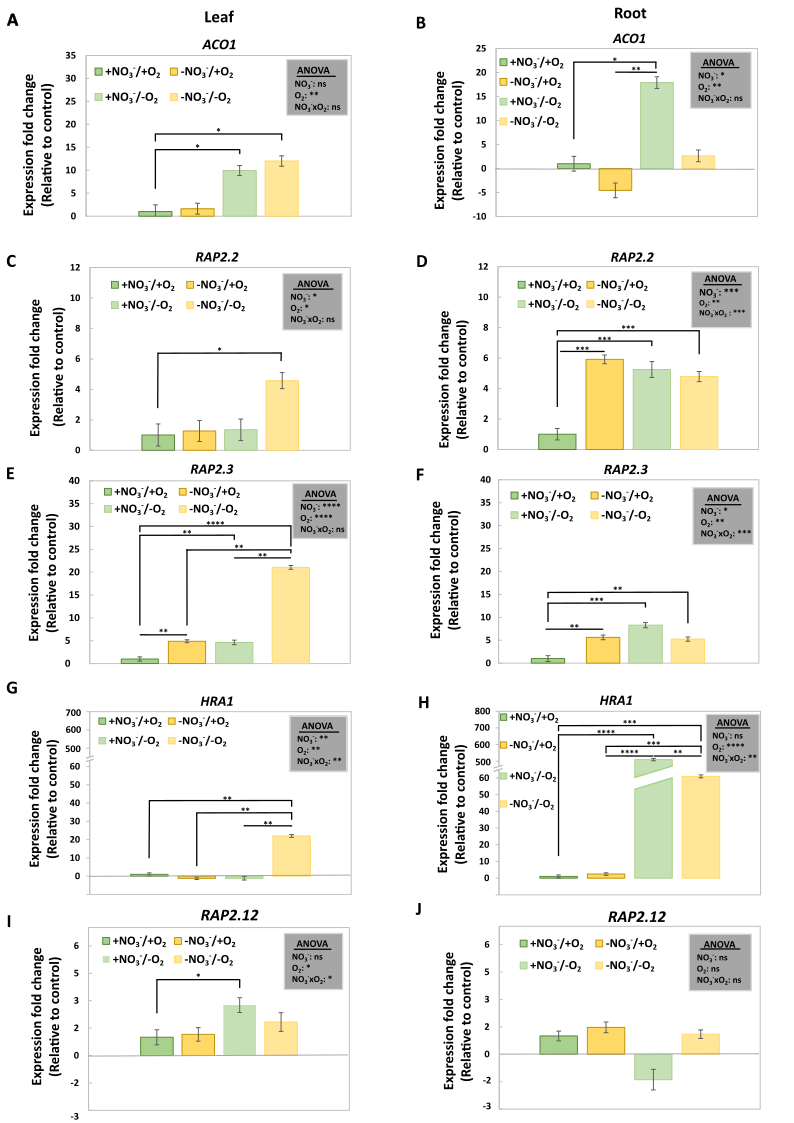
**

**
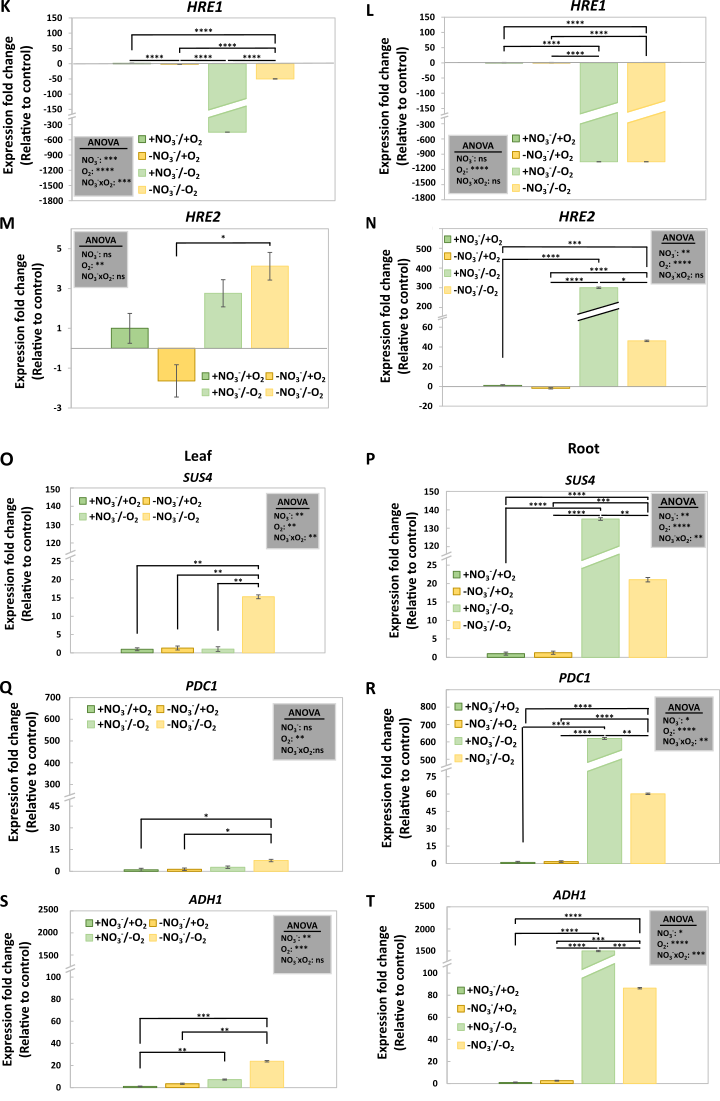

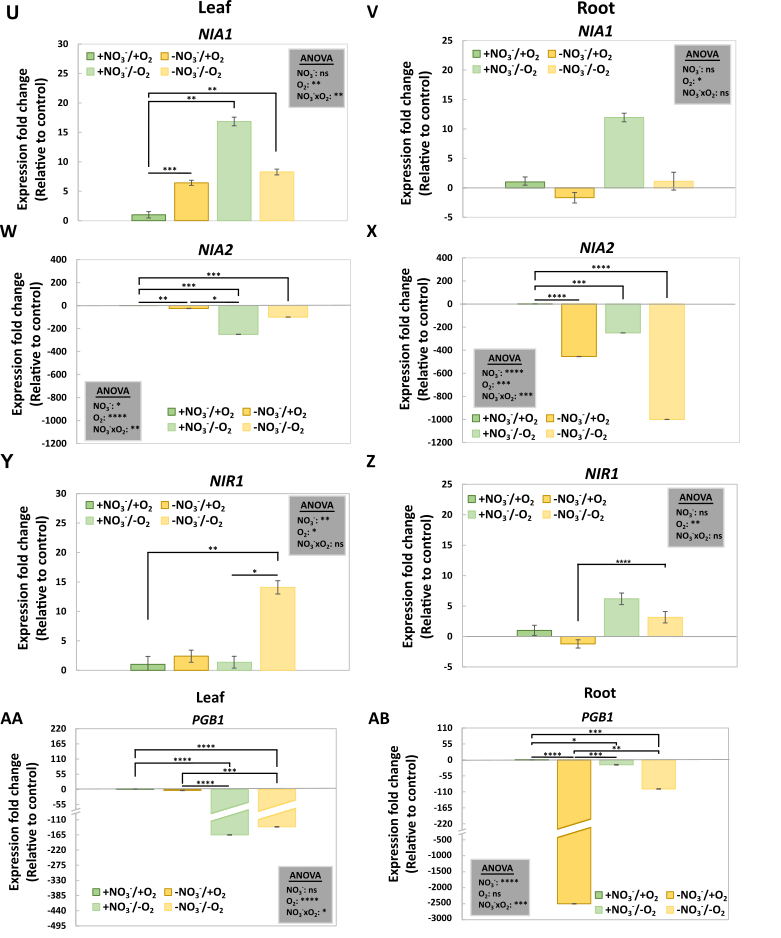
**

**
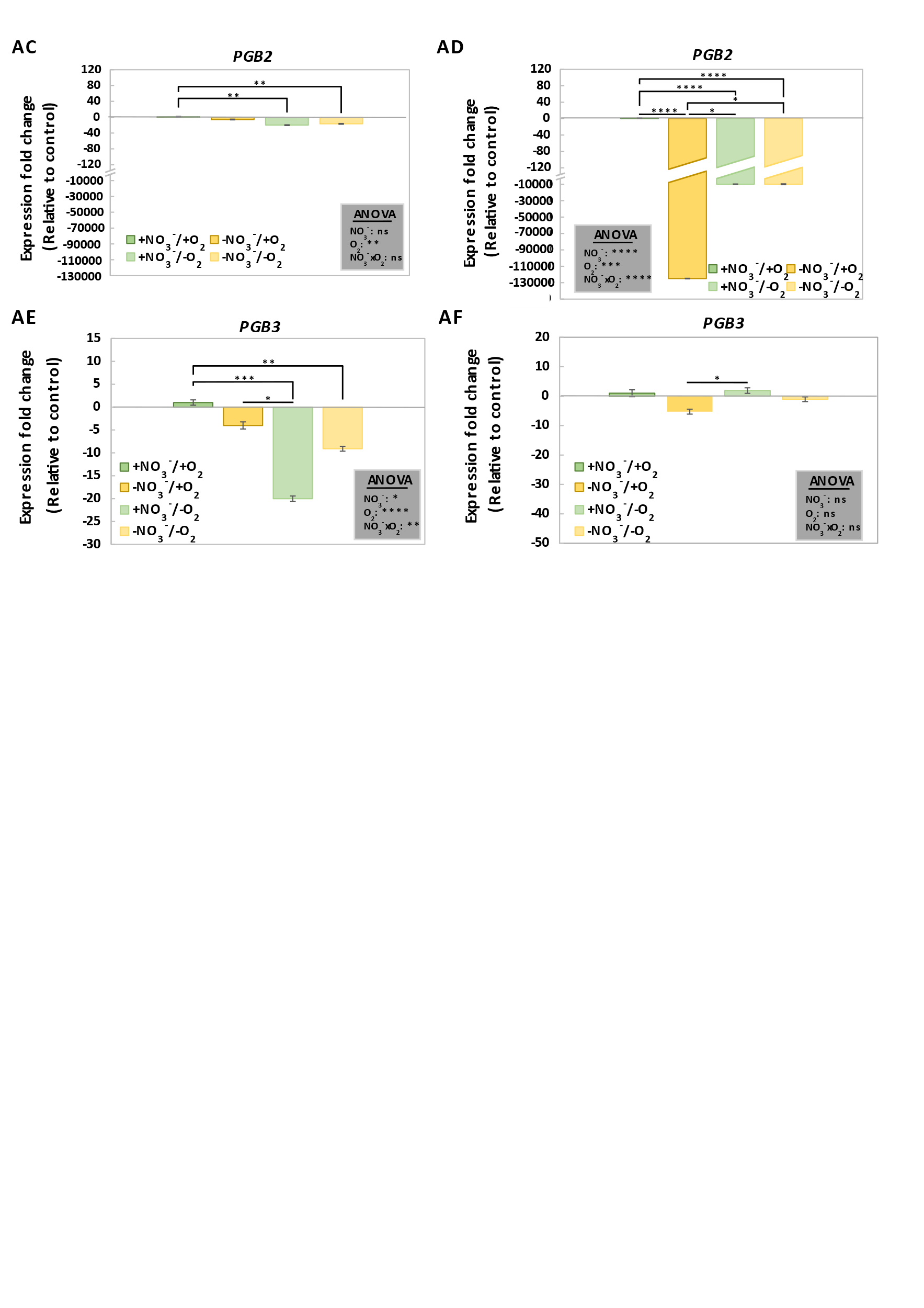
**
